# Web-Based Self-management Program (SPACE for COPD) for Individuals Hospitalized With an Acute Exacerbation of Chronic Obstructive Pulmonary Disease: Nonrandomized Feasibility Trial of Acceptability

**DOI:** 10.2196/21728

**Published:** 2021-06-11

**Authors:** Linzy Houchen-Wolloff, Mark Orme, Amy Barradell, Lisa Clinch, Emma Chaplin, Nikki Gardiner, Sally J Singh

**Affiliations:** 1 Centre for Exercise and Rehabilitation Science University Hospitals of Leicester NHS Trust Leicester United Kingdom; 2 Department of Respiratory Sceinces University of Leicester Leicester United Kingdom

**Keywords:** COPD, telehealth, digital health, internet, rehabilitation, quantitative, qualitative, exercise

## Abstract

**Background:**

Hospital admissions due to the acute exacerbation of chronic obstructive pulmonary disease (AECOPD) are costly for individuals and health services. Pulmonary rehabilitation (PR) is known to reduce hospital readmissions when delivered after hospitalization, but the uptake and completion of PR following hospitalization remains poor (<10% of those eligible in the UK audit data). A web-based platform of the SPACE (Self-management Program of Activity Coping and Education) for COPD (chronic obstructive pulmonary disease) has previously shown promising results in patients with stable COPD but has not been tested following an AECOPD.

**Objective:**

This study aims to assess the feasibility and acceptability of a web-based self-management program.

**Methods:**

A nonrandomized feasibility study for patients with confirmed AECOPD who were deemed web literate was conducted. All patients consented during their hospitalization and received access to the website following discharge in addition to usual care. The program aims to facilitate patients to better understand and manage their condition through education and home-based exercises. Participants were asked to complete the Bristol COPD Knowledge Questionnaire at baseline and after 6 months. A total of 14 participants were also interviewed (n=8 completers; n=6 noncompleters) regarding their experiences with the web-based program and trial. The interviews were analyzed using thematic analysis.

**Results:**

In total, 2080 patients were screened for eligibility, of which 100 patients (age: mean 71.2 years, SD 9.3 years; male: 55/100, 55%; forced expiratory volume in 1 second/forced vital capacity ratio: mean 0.46, SD 0.14; pack-years: mean 50.2, SD 31.0; current smokers: 35/100, 35%) were recruited (4.8% of those screened). The main reason for ineligibility was a lack of web literacy (1366/1980, 68.98%). In total, 18% (18/100) of patients had completed the web program by 6 months, with others still registered in the program (27/100, 27%), and more than half did not register (55/100, 55%). There was a mean change in Bristol COPD Knowledge Questionnaire scores at 6 months of 7.8 (SD 10.2) points. Qualitative interviews identified three main themes: preparing for, engagement with, and benefits of the study and program. A total of 57% (57/100) accepted a referral to PR on discharge and 19% (19/100) had completed the program after 6 months.

**Conclusions:**

On the basis of the challenges of recruiting, retaining, and engaging participants in a web-based self-management program, it is not a feasible approach to roll out widely. This study acknowledges that this is a challenging time for patients with an AECOPD to engage in exercise and self-management education. However, for patients who were able to engage in such an intervention, the completion rate of PR was double the previous audit estimates from the United Kingdom, disease knowledge improved, and the intervention was of value to patients.

**Trial Registration:**

ISRCTN Registry 13081008; https://www.isrctn.com/ISRCTN13081008

## Introduction

### Background

Hospital admissions for the acute exacerbation of chronic obstructive pulmonary disease (AECOPD) represent a huge burden to the individual in terms of muscle dysfunction, breathlessness, and inactivity [1]. Furthermore, the AECOPD is costly to health services, particularly when an inpatient stay is required [2,3]. Pulmonary rehabilitation (PR) is a high-value and cost-effective intervention that is safe and improves exercise capacity and quality of life [4], outplaying pharmacotherapy and telehealth [5], and may offer a survival advantage for those who complete the program [6]. As such, PR is recommended by national and international guidelines soon after an exacerbation [7,8].

Despite this guidance and the established benefits of PR, access to, uptake to, and completion of postexacerbation rehabilitation are very poor. In the United Kingdom, less than 10% of all hospital discharges for the AECOPD completed posthospitalization PR [9]. More recently, the UK National Audit found that only 3% of the audit caseload is for postexacerbation rehabilitation, with the rest attributed to patients with stable respiratory disease. We also know from data from the United Kingdom that often the suitability for rehabilitation is not assessed at discharge by the clinician in as many as 44% of cases [10]. Therefore, many potentially suitable patients are missing from this valuable intervention. This problem is not unique to the United Kingdom; indeed, recent figures from the United States suggest that only 1.9% of patients hospitalized for chronic obstructive pulmonary disease (COPD) exacerbation receive PR within 6 months of discharge. The rate of uptake varies widely according to geographic region and ethnicity [11].

The reasons for nonuptake and poor completion are underpinned by complex reasons; some are related to the organization and system of delivery and others to patients’ individual choices [12,13]. A key problem in the postexacerbation phase is that patients feel too unwell or breathless to attend a hospital or community program [14]. At the time of starting this study, there had been no randomized controlled trials (RCTs) of interventions to increase the uptake of early rehabilitation following exacerbation. One quasi-randomized study (n=115) with a high risk of bias indicated greater program completion and attendance rates in participants allocated to PR alongside a tablet computer (support for exercise training) compared with controls (PR only [15]). Other studies have explored the role of changing the timing of postexacerbation rehabilitation by moving this into the periexacerbation phase (inpatient [16]) or by delaying the start for 7 weeks [17] or 6 months [18]. These initiatives have had little benefit over control conditions, and recruitment was a challenge for delayed studies [17,18]. One feasibility study has also looked to reduce sedentary time in those hospitalized with an AECOPD using wearable technology (a vibration prompt to move at intervals throughout the day) for 2 weeks postdischarge [19]. Collectively, patients responded to 32.6% (106/325) of vibration prompts from the waist-worn device. Qualitative interviews indicated that being unwell and overwhelmed after an exacerbation was the main reason for not engaging with the intervention, and retention in this study was poor (52%).

To address the problem of uptake, over the past 10 years, we have developed home-based alternatives to attending a traditional center or community-based PR. These remote models have become increasingly relevant in the era of COVID-19, as the social distancing measures taken in many countries to suppress transmission of SARS-CoV-2 have had an immediate and profound effect on the provision of PR services [20]. SPACE (Self-management Program of Activity Coping and Education) for COPD is a self-management program of activity, coping, and education, which was coproduced as a 4-stage manual by health care professionals and patients [21]. In a series of studies, it has been shown to improve symptoms and exercise tolerance above usual care control groups and is noninferior to PR for improvements in quality of life [22,23]. When delivered in a hospital, SPACE for COPD was able to improve the quality of life and readiness for home above the control [24]. More recently, we have transitioned the SPACE for COPD program to a web-based format because there is a real ambition for health care services to engage with new technologies [25]. This has been driven by data showing that internet use in the >75-year age group is rising rapidly, closing the gap in the younger age groups [26]. Clearly, these would be the target age groups for rehabilitation interventions. To this end, we tested the web-based version of SPACE for COPD in secondary care and found the approach to be feasible and acceptable when compared with standard rehabilitation [27]. However, we have not yet tested the web-based version of SPACE for COPD in an acute, hospitalized population. We also know that patients are very inactive upon hospital discharge [28]; therefore, home-based solutions that act as a stepping stone to outpatient PR may be warranted. Otherwise, there is a drastic increase in patient expectations.

### Objective

The primary aim of this study is to assess the feasibility and acceptability of a web-based program for individuals hospitalized with COPD exacerbation.

## Methods

### Population

This was a single-center, nonrandomized feasibility study. All patients admitted with an exacerbation of COPD to Glenfield Hospital, Leicester, were screened for eligibility by the specialist COPD nursing team and given at least 24 hours to consider the information. We included patients with an email address who were web literate (used a tablet, PC, laptop, or device at least once per week). To assess this, we asked patients about the types of devices used, the time spent on the web, and the types of web-based activities for each patient (eg, emailing, internet banking, and web-based shopping). A decision on web literacy was at the discretion of the recruiting clinician. The program was predominately delivered via a tablet. Patients could use their own device or borrow an Android tablet, for instance, if they usually borrowed a family member or did not have access for the duration of the study. Borrowed devices were locked (SureLock software, 42Gears Mobility Systems Limited), other than for access to the SPACE for COPD website. People with significant neuromuscular or cardiovascular comorbidities limiting physical activity (typical exclusion criteria for PR) and those who were unable to read and write in English were excluded. Currently, the website is available only in the English language.

### Intervention

Patients were given access to the SPACE for COPD program as an inpatient. A passport card with log-in information and staff contact details and a user manual were given to the patient on discharge along with a verbal introduction to the program (alongside viewing this on a tablet) by the COPD nursing team.

SPACE for COPD is an interactive web-based program that offers a comprehensive package of exercise and self-management education. The program was structured to guide the user through four stages, each of which has specific tasks that the user needs to achieve before progressing to the next stage. Tasks included creating and updating their own short-term goals, completing knowledge tests on COPD and exercising safely, and reading or watching videos on specific topics, such as inhaler techniques or healthy eating. The program was described in detail by Chaplin et al [27]. In stage 2, patients were asked to record their aerobic walking exercise, and for this study, we devised a symptom diary that linked to the patients’ individual exacerbation action plan. The web-based program usually takes approximately 11 weeks to complete for patients with stable COPD [27], although this patient cohort had access for 1 year, to promote long-term behavior change and maintenance. However, the outcomes were assessed at 6 months. Special features of the program include videoconferencing (where patients could have a live consultation with the COPD nursing team at an allocated time), a moderated blog section (where patients could share their experiences with others), and an *ask the expert* facility (to email the COPD nursing team). The *ask the expert* emails were monitored by the specialist COPD nursing team during working hours (Monday to Friday, 8 AM-4 PM). Prompts to log on to the website and record activity were automatically generated by the program and sent via email if patients failed to record activity for 7 consecutive days. Patients also received a telephone call from specialist COPD nurses within the first 5 days following discharge (as is usual care).

Usual care, including referral to and attendance at PR, was not affected by this trial; patients received a telephone call from the COPD nursing team within 5 days of discharge and had a scheduled follow-up appointment with a consultant within 3 months of discharge.

### Outcomes

The primary outcome was the feasibility of the intervention (uptake to the intervention: percentage of patients recruited out of the total number screened and completion rates of the Bristol COPD Knowledge Questionnaire [BCKQ]). This questionnaire was chosen because the authors felt that it was the least likely to be influenced by illness and natural recovery following the AECOPD. The secondary outcomes were the acceptability of the intervention and trial (qualitative interviews), intervention engagement (web usage statistics: number of log-ins and use of web features captured directly from the administrator section of the website), and uptake to outpatient PR (uptake and completion rates in those referred). All outcomes were assessed at baseline (in hospital upon enrollment to the study) and 6 months following enrollment in the study, regardless of whether the patient had engaged with the web-based program or PR during the 6-month period.

### Analysis

#### Quantitative Analysis

Data are described as mean (SD), median (IQR), or frequency (%), as appropriate. No inferential statistics were obtained owing to the feasibility nature of the trial. As is convention with feasibility studies, a formal sample size is not required [29]. A total of 100 patients were recruited.

#### Qualitative Analysis

To measure patients’ views on the acceptability of the web-based program and the study, qualitative interviews were conducted with completers and dropouts on a purposive sampling basis (completer and noncompleter interview schedules are shown in Multimedia Appendices 1 and 2 respectively). Patients who agreed to be contacted for an interview were approached following the 6-month study time point, as it was anticipated that most patients would have accessed and/or completed the web-based program by this point.

The interviews were conducted by the qualitative researcher AB, who was relatively naïve to the web-based program and study processes. After each interview, the researcher wrote reflective and methodological notes to assist the analytic process and enhance the rigor of the results.

The interviews were audio recorded and transcribed verbatim using an external source. Analysis of the interviews was conducted manually using the thematic analysis framework by Braun and Clarke [30]. The six phases of the framework were followed by AB, LHW, SJS, and MO, who independently coded the interviews. These phases included data familiarization, data coding, searching for themes, reviewing themes, defining themes, and composing the narrative. Agreement of themes was made by the four coders.

## Results

### Primary Outcome

A total of 2080 patients were screened over 2 years (May 2015 to September 2017) to obtain a sample of 100 patients. The proportion of patients recruited as 4.8% of those screened (100/2080). The predominant reason for exclusion in approximately (1366/1980, 68.98%) of cases was that patients were not web literate or did not have an email address. Table 1 provides the reasons for exclusion or nonuptake.

**Table 1:** Reasons for exclusion or nonuptake (N=1980).

Reason	Patients, n (%)
Not web literate, no email address	1366 (68.98)
Unwilling	297 (15)
Comorbidities precluding involvement in the study	238 (12.02)
Has done pulmonary rehabilitation or SPACE^a^ previously (did not want to do it again)	40 (2.02)
On another research study	20 (1.01)
Unable to read English	19 (0.95)

^a^SPACE: Self-management Program of Activity Coping and Education.

Of the participants recruited, the mean age of participants was 71 (SD 9) years; they had severe disease and a mean smoking pack-year history of 50.2 (SD 31.0) years (Table 2). There was a good split between male and female participants: 55% (55/100) males, 35% (35/100) were current smokers, and most had at least one other comorbidity (93/100, 93%).

**Table 2:** Baseline characteristics of recruited participants.

Variables	Value, mean (SD)
Age (years)	71.2 (9.3)
BMI	28.1 (9.8)
FEV_1_^a^/FVC^b^	46.2 (13.9)
FEV_1_ (% predicted)	44.8 (18.3)
Pack-years	50.2 (31.0)

^a^FEV_1_: forced expiratory volume in 1 second.

^b^FVC: forced vital capacity.

### Secondary Outcomes

#### Disease Knowledge

The change in the BCKQ score was 7.8 (SD 10.2) points, an increase of 21% (prescreening score: mean 37.1, SD 9.5; postscreening score: mean 44.9, SD 9.4). This was done in 42 patients who returned the questionnaires at 6 months.

**Figure 1:**
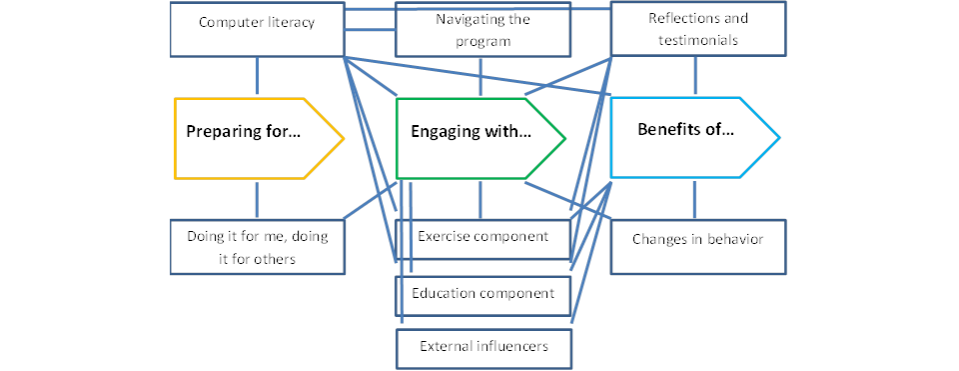
Qualitative thematic map.

Qualitative interviews with a sample of 14 patients (n=8 completers; n=6 noncompleters) identified three themes, with a total of eight subthemes. The relationships between the themes are presented in Figure 1. The identified themes were *preparing for*, *engaging with*, and *benefits of* the web-based program.

#### Preparing for: Doing It for Me

The patients described a number of reasons why the web-based program incited their internal motivation to engage with a telerehabilitation program. Some studies have described the fear they experienced from a recent COPD exacerbation as a catalyst:

...when you’re recovering from something that frightens you to death, you say yes to everything. So that’s why I said yes.

Participant 69, completer

They also felt that the program offered an opportunity to learn how to manage their condition more successfully through the ongoing support of health care professionals:

I wanted to learn more and to see if there was anything that would be beneficial to the breathing obviously. Because I mean we know that the rehabilitation works. I can vouch for that. Unfortunately it has a very short shelf life unless you continue it. So I was hoping that just get information because there really isn’t that much information. Your own GP doesn’t seem to have, I mean I’ve got a fantastic GP, but he hasn’t got a lot of information on COPD. If you have a problem, he’ll refer you to the respiratory nurse who comes about once a year.

Participant 39, completer

The appeal of a home-based program was also evident among patients. They felt it offered them the ability to complete the program at their own pace, and this flexibility allowed them to fit it into their established routines:

...she said you can do it at home on your laptop and in my own time. I haven’t got to go out the house, well I’ve got to go out the house to walk.

Participant 100, noncompleter

#### Preparing for: Doing It for Others

A subset of patients described external influences as factors that motivated them to engage in the program. Some felt that the reassurance health care professionals offered was what they needed to engage with the program. Others felt that their families were instrumental in encouraging their engagement with the program. One patient described how the support of her family gave her the confidence to engage:

but I thought—like my children said to me, if you’re not doing that mam, what are you going to be doing. Thought well, yeah, they’re right, why shouldn’t I do it.

Participant 52, completer

For another, the program was viewed as an opportunity to share the learning materials with their loved ones in an effort to improve both their health:

...I was hoping that at the end of it I would have a bit more information as to - because my missus had got COPD as well and there was something we could learn from it.

Participant 39, completer

#### Preparing for: Computer Literacy Skills and Suitability

Overall, patients felt that their computer literacy skills were vital to their ability to engage with the program. Despite the assessment of computer literacy skills before enrollment in the study, some patients felt that their age or generation acted as a barrier to their ability and many lacked confidence in their skills:

I mean for you and for your generation, you don’t even think about it do you, you just do it. For us, we never had it. It was chalk and slate!

Participant 39, completer

#### Engaging With: Navigating the Program

All patients felt that the program was complex in nature and learning to navigate it felt like a steep learning curve. Some felt it was too steep a learning curve for their computer literacy skills, which resulted in reduced motivation to engage with the program or, for some, to disengage from the program entirely:

...you spend more time on the computer than walking. So that defeated the object with me anyway.

Participant 100, noncompleter

To tackle this issue, some patients sought help from family members or health care professionals but still found the interface challenging and at times overwhelming:

It wouldn’t let me in. I tried it on Safari and it kept refusing me and then I finally got on...And I just couldn’t get on with it. I got it on in the end with the help from my daughter and then all the questionnaires, never ending, just one page after another...what’s your lung capacity? How do I know? What is it when you breathe in, what is it when you breathe out?—terrible!

Participant 100, noncompleter

For those patients who persevered with the program, they felt self-discipline, self-motivation, and allocating time to learn how to navigate the program were essential to their progression:

As I say, after about three to four weeks I was very confident or confident, I’ve never been very confident at any of it, confident, but I must be honest it did take me about that long before I felt happy doing it. You know, I did it and I’d sit there for ages doing it and my missus would say to me have you done it yet? But, you know, you’d persevere....

Participant 39, completer

Some patients expressed frustration due to the interaction they had with the program, particularly the email reminders to exercise daily as they felt it lacked the acknowledgment of their individual internal and external barriers to exercise:

I found myself getting a bit wound up when I’d not carried out certain tasks to the satisfaction of the, you know, the interaction was you haven’t exercised this week. No I haven’t because I’ve not been well!

Participant 69, completer

Other patients embraced the exercise reminders and enjoyed the *little nudge*, as it helped them develop exercise habits:

I found it very good because you’re doing it at your own pace. And it wasn’t excessive and it just gave you that little nudge in the back of your mind. You’d look at your emails and it would come up SPACE and you’d think oh ah. It were more like, not a guilt trip, that’s wrong, but it just give you that little nudge and you think. But once you got into it, you know, you did it automatic which was a good thing.

Participant 39, completer

A further facility to initiative interaction with health care professionals was the teleconferencing option, but this was not used by any participant. Some patients felt it was too advanced for them, others felt it lacked a personal approach from staff, and some were unaware of this option:

I think I did see it, but that, I’m not, I’m better off person to person, not person to screen.

Participant 78, noncompleter

#### Engaging With: Exercise Component

For patients who reached the exercise component of the program (stage 2), many felt goal setting was beneficial, as it motivated them to exercise more, which in turn had a positive impact on their quality of life:

Yeah, my breathing is better when I’ve done the exercises, when I’ve done the walking. It don’t feel at the time, I’m gasping like hell. Later in the day you feel the benefits of it. It always clears your lungs out, it clears your lungs doing the walk. And I enjoy that.

Participant 100, noncompleter

The daily recording and monitoring of the patients completed also helped some to identify patterns in their exercise behavior, and from these patterns, they could identify earlier onset of COPD exacerbations:

And what was interesting with that was if you were honest and put it down on the programme, you started to see a pattern building up. And I don’t know what the pattern meant or whatever but you could see when you were coming up to, you know, one of the and you needed the antibiotics, you could see it before.

Participant 39, completer

However, patients felt that their ability to exercise was restricted by factors outside their control. For example, some did not prioritize exercise if they felt unwell or if the weather was deemed inclement. However, one patient who faced this scenario did not let this deter him from exercising:

I go on my bike when I can’t go out then I’ll stand and do a few knee exercises, stretches and bending and things.

Participant 100, noncompleter

#### Engaging With: Education Component

Overall, patients felt that the education component developed their understanding of COPD and increased their knowledge and skills regarding self-management. They felt that the program highlighted the importance of exercise and gave them important practical skills:

The breathing, the way that you breathe. The necessity to exercise is the thing that comes out of all of it, even if it’s only small amounts. And if you can’t hit what you set yourself to do, don’t beat yourself up with it. Just do it, do the best you can, and that’s the thing that came out of it. If you can’t do what you want to do on that particular day, do what you can do.

Participant 39, completer

Some patients felt that the program also helped them understand the relationship between COPD and mood. This understanding helped them recognize their feelings as a natural reaction to living with a long-term condition:

...one of the other things that came out about it was things like getting depressed. I don’t tend to get depressed, I tend to get fed up with it all and you think is this it, whatever? Which is again very normal but you don’t know that and you think what’s happening? They tell you that.

Participant 39, completer

#### Engaging With: External Influencers

Some patients felt that their ability to complete the program was affected by factors outside of their control, which reduced their motivation and level of discipline. Exacerbations of COPD, comorbidities, and becoming a caregiver for a loved one were all described as reasons for noncompletion:

So once I got used to it I didn’t really have a problem with it. And I followed that religiously. And then we hit a snag, but it was nothing to do with the course. My missus had to go into hospital for three months because she had a brain haemorrhage. So when she came out, she still needed, we needed to put the time in to get her back to normal again, which was three, four, five months. So that then took a little bit of a backseat and I found then I couldn’t quite catch up.

Participant 39, completer

#### Benefits of: Reflections and Testimonials

Patients who engaged in the program felt it gave them a focus and drive to take control of their health. Patients felt that the program helped them reassess their illness perceptions, which, in turn, made them feel less self-conscious, more confident in self-managing their COPD, and gave them hope for the future:

I feel in control of it. Instead of it controlling me

Participant 52, completer

#### Benefits of: Changes in Behavior

Some patients felt that the program inspired them to challenge their health behaviors and encouraged them to make positive changes. Patients attributed increases in daily exercise to the program, and others found affirmation for smoking cessation:

[I’ve learned] Never to smoke again.

Participant 88, completer

Program completers and noncompleters all felt that the program helped them find enjoyment in exercise, and this encouraged a continuation of exercise following program completion. To facilitate this, some patients purchased exercise equipment, downloaded fitness apps, recorded their daily exercise, and began to embed exercise into their daily routine. This increase in exercise meant that some participants were now more capable of carrying out their daily activities:

I managed to do all my garden last year instead of having a gardener...I can now have a shower and I wash everywhere down.

Participant 52, completer

#### User Statistics

None of the participants used videoconferencing or blog facilities. The median number of log-ins per person was 14 and emails to the team was 1. Patients generally recorded more than one goal throughout the course of the program, an average of 3.6 (SD 2.4) per person, and an average of 16.5 (SD 32.7) of the exercise sessions were recorded.

At 6 months, 18% (18/100) of participants had completed the web-based program, 27% (27/100) were still registered, and 55% (55/100) had not registered, despite prompts by email and a phone call from the nursing team within the first 5 days following discharge. The fact that one-fourth were still registered at 6 months suggests that the acute population may need longer to complete the program compared with a stable population.

#### Uptake to PR

Of the total 100 participants in the sample, 57 accepted a referral for rehabilitation. Of these, 47 were assessed, and 35 started a program; 19% (19/100) of the total population completed either a hospital or community outpatient rehabilitation program.

## Discussion

### Principal Findings

On the basis of the challenges of recruiting, retaining, and engaging participants in the SPACE for COPD web-based self-management program, it is not a feasible approach to roll out widely. In the face of organizational pressure to provide health information on the web and despite statistics suggesting otherwise, digital literacy in this group was lower than anticipated, as evidenced by the low number of patients deemed to be web literate.

Engagement in the program was poor. At 6 months, only 18% (18/100) had completed the web-based program, 27% (27/100) were still registered, and 55% (55/100) had not registered, despite prompts via email and phone calls from the nursing team. The fact that one-fourth of participants were still registered at 6 months perhaps indicates that an acute population takes longer to complete the program compared with a stable population (stable—complete within 11 weeks [27]). The lack of engagement is surprising, given that patients willingly signed up to the study; it may be that patients overestimated their information technology skills. In a recent qualitative study by Slevin et al [31], patients reported a willingness to take a more active role in self-management using digital health technologies. They perceived digital health technologies as potentially enhancing their self-management skills *by improving self-efficacy and engagement and by supporting health care professionals to practice preventative care provision*. However, we are aware that, although web usage among people aged >75 years is increasing [26], this may range from simply sending an email through to more complex tasks such as web-based banking or navigating websites. For instance, older patients, those with a lower socioeconomic status and those with more severe health needs are less likely to use technology or to handle eHealth-based tasks [32]. This is of concern given that the PR community has been challenged to rapidly provide innovative and alternative ways of delivering rehabilitation in the face of the COVID-19 epidemic, despite the evidence that the efficacy of digital self-management and rehabilitation programs in COPD is uncertain [33]. It may be that for this population, additional training on using the website will be required with a competency assessment built in to increase uptake, engagement, and completion.

The patient interviews revealed a strong divide among participants’ willingness to engage in the program. Those who engaged demonstrated competent computer literacy skills, attributed more value to the program, experienced more external motivation from it, and were able to use it flexibly to adapt to their health and other life events. Those who did not engage with the program experienced greater difficulty in navigating the website, which they felt reduced their internal and external motivation to complete the program. This finding was also true of the TELEKAT (Telehomecare, Chronic Patients and the Integrated Healthcare System) study [34], in which severe to very severe patients with COPD expressed commitment to the program if they had a prior interest in telehealth or new technologies. However, this commitment did waiver when patients experienced COPD flare-ups. Therefore, it would be helpful to identify patients who would benefit from a different approach, particularly around the time of an exacerbation.

These results suggest that patients’ exercise behavior was influenced by their intention to exercise, and this was further negated by their internal and external motivation, their opportunity to exercise, and their physical ability to exercise. These findings support the *capability, opportunity, motivation, and behavior* model of behavior change [35], which describes how behavior is driven by intention, which is further negated by motivation, opportunity, and capability. The model predicts that when one or more of these factors are reduced, the intention to carry out the behavior is also reduced.

However, in those recruited to this study, the completion rate of PR was 19%. This is double the previous audit estimates from the United Kingdom and significantly higher than the 1.9% proposed recently from the United States [9,11]. In addition, disease knowledge improved by 21% in this cohort. Although there is no accepted minimum clinically important difference for BCKQ, typical changes following outpatient PR are in the region of 18% [36].

Therefore, we would conclude that web-based strategies may be a viable stepping stone to postexacerbation PR in those able and willing to engage with the program.

### Limitations

The main limitation of the study is that it was not an RCT; therefore, the effects of natural recovery were not considered. However, we chose to look at disease knowledge as a secondary outcome (rather than an outcome such as exercise tolerance or muscle strength), which is less likely to be influenced by exacerbation recovery. Furthermore, as this was a feasibility study, we did not intend to infer clinical effectiveness. We accept that postal returns of questionnaires are not the most reliable way to return data, and we followed this up with a phone call prompt to increase data completion. It may have been interesting to examine the differences in outcomes such as hospital readmission and uptake to PR in a matched usual care–only cohort (ie, those not receiving the web program), but we did not seek ethical approval to do this.

This was also a single-center study, which may introduce selection bias and decrease the generalizability of the findings to other settings. Further concordance of qualitative themes between the four coders using statistical methods (eg, κ) would have strengthened these data further.

As stated previously, patients or recruiting clinicians may have overestimated the patients’ ability to navigate the web program. This was despite strictly adhering to our screening criteria (must be web literate and must have a valid email address), a validated digital literacy measure would have been useful to aid screening.

The timing of enrollment in the study and introduction of the intervention were pragmatic and not standardized. We tried to do this as close to discharge as possible, but this was not always easy to predict in practice. We appreciate that a hospital admission may not be the best time for patients to be receptive to new information. In particular, it appears that cognitive function is specifically impaired during exacerbation but may recover [37]. Therefore, shifting our intervention introduction to a later time point following discharge may have been preferable.

Although the completion of postexacerbation rehabilitation in this study is impressive, it is of course with the caveat that these were self-selecting, enthusiastic research participants rather than *all comers*. Therefore, it is likely that these participants had a personal interest in self-management of their disease. In addition, because the web-based program acted as a graduated transition into outpatient PR, there may have been participants who stopped using the web-based program when they started PR or vice versa (ie, used the web program and did not attend PR). We have not been able to tease out these nuances in our data.

### Comparison With Prior Work

Several gaps still exist in the literature on the topic of increasing access to and participation in PR. There may be instances where completion rates for PR are buried within manuscripts as a secondary outcome, but as it is not the primary outcome, it is unlikely to change guidance. This may be because we have no idea what an acceptable level of uptake or completion would be; therefore, studies tend to choose primary outcomes based on the quality of life or exercise measures, where we already have a wealth of data or minimum clinically important difference values. Therefore, high-quality research is needed to review complex interventions with uptake or completion as the primary outcome. In addition, we might assume that many centers have adapted their programs in pragmatic service improvement initiatives to increase acceptability without robust testing of these methods. This would account for the gaps in the literature.

Since we have completed our study, one RCT has been published, which evaluated the effect of a co-designed (by patients and health care professionals) education video as an adjunct to usual care on posthospitalization PR uptake. PR uptake was 41% and 34% in the usual care and intervention groups, respectively (*P*=.37), with no differences in secondary (PR referral and completion) or safety (readmissions and death) outcomes. Unfortunately, 40% (6/15) of participants interviewed did not recall receiving the video.

Other digital health apps or telerehabilitation have been reported in the literature for COPD populations [38,39]. Of note, the myCOPD app is a web-based 6-week rehabilitation program, which was found to be noninferior to a conventional PR delivered in face-to-face sessions in terms of the effects on walking distance and symptom scores (COPD assessment test) at 7 weeks [38]. The program was safe and well tolerated; however, it is worth noting that the population recruited was milder with fewer comorbidities than is usually reported in PR studies and that adherence to exercise sessions was slightly lower in the web-based group than in the face-to-face sessions per week [38]. The same app has been routinely offered to stable patients in Hammersmith and Fulham Respiratory Clinics (London, United Kingdom). Although two-thirds of patients were eligible to use the app (64%, 163/253), this has not translated into uptake (56%); 15% (297/1980) of our population declined to take part or engage with the PR program (10%), and this challenges the assumption that this digital app can be delivered as a suitable alternative to standard PR [40]. It is not clear what engagement referred to in this report, but if it was the completion of the app program, then a 10% noncompletion rate is disappointing and concurs with poor completion of the web program at 6 months in this study (18/100, 18%). Recently, Polgar et al [41] assessed the digital habits of patients referred to PR during the COVID-19 pandemic. There was significant heterogeneity in access to and confidence in using the internet, with 31% having never previously accessed the internet, 48% confident using the internet, and 29% reporting no interest in accessing any component of PR through a web-based application [41].

A recent qualitative work by Janaudis-Ferreira et al [42] has begun to *set the stage* to design a more acceptable PR program following an exacerbation of COPD. In this study, one-on-one interviews were conducted to explore the views of 13 patients and 11 health care professionals on PR after the AECOPD and how participation could be enhanced. Four main themes were identified: (1) uncertainty regarding the timing of PR; (2) tailored and flexible manner to deliver PR programs with a gradual start; (3) education for all; and (4) logistical, disease-related, and psychological barriers. Theme 2 is particularly interesting and aligns well with this study, as the web-based intervention may be thought of as a *bridge* to start a formal PR program. Theme 4 chimes with some of the participant quotes from our qualitative work.

### Conclusions

On the basis of the challenges of recruiting, retaining, and engaging participants in this web-based self-management program, the SPACE for COPD web-based self-management program is not a feasible approach to roll out widely following an AECOPD. It appears that the COPD population may not be equipped and ready for digital self-management interventions following an AECOPD, without additional training or support. This work acknowledges that this is a challenging time for patients with an AECOPD to engage in exercise and self-management education. However, for patients able to engage with such an intervention, the completion of PR was double the previous estimates from UK audit or research data, disease knowledge improved, and it encouraged positive behavior change and was of value to patients. Therefore, with further refinement, web-based (or other home-based) strategies may be a viable stepping stone for PR. Identifying the patients most likely to benefit from such strategies is warranted, particularly if social distancing measures for COVID-19 are to continue.

## Appendix

Multimedia Appendix 1 Completers' interview schedule.

Multimedia Appendix 2 Noncompleters' interview schedule.

## Abbreviations

AECOPD: acute exacerbation of chronic obstructive pulmonary disease
BCKQ: Bristol COPD Knowledge Questionnaire
COPD: chronic obstructive pulmonary disease
PR: pulmonary rehabilitation
RCT: randomized controlled trial
SPACE: Self-management Program of Activity Coping and Education
