# Supplementary material for: Web-Based Self-management Program (SPACE for COPD) for Individuals Hospitalized With an Acute Exacerbation of Chronic Obstructive Pulmonary Disease: Nonrandomized Feasibility Trial of Acceptability
Source: JMIR Mhealth Uhealth. 2021 Jun 11;9(6):e21728. doi: 10.2196/21728 (PMC8235284; doi:10.2196/21728)
Supplement: Multimedia Appendix 1 [file mhealth_v9i6e21728_app1.doc]

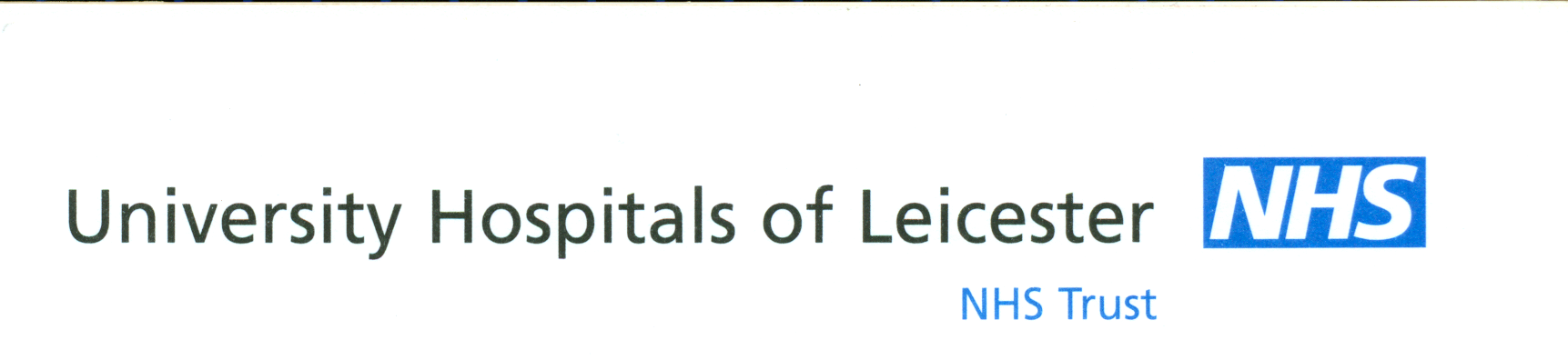


Glenfield Hospital

Groby Road

Leicester

LE3 9QP

Tel: 0300 303 1573

Fax: 0116 258 3950

Minicom: 0116 287 9852

**Follow-up Interview – Topic Guide for COMPLETERS**

**InterSPACE – COPD Self-Management Programme**

InterSPACE: Feasibility of an integrated Telehealth and Self- Management programme for individuals hospitalised with an exacerbation of COPD.

- **Gain consent and introduction**
  - *Introduce you, name and role*
  - You have agreed to take part in an interview which is an informal chat with me. This will
    be about web-based InterSPACE study and how you found this web-based self-management programme. It is a chance for you to tell me anything that you think is relevant.
  - Do you have any questions so far?
  - Are you happy for me turn on the Dictaphone to record the interview as it takes place?
  - For the benefit of the tape it is …day…..time and present there is me, …… (the patient)
    and …….
- **Withdrawal**
  - If anything we speak about today does make you feel uncomfortable you are free to
    not answer a particular question, you can ask for the recorder to be switched off to
    resume the interview after a short break or you can ask to terminate the interview all
    together at any point.

Contd….

- **Participation in the InterSPACE study**
  - What influenced your decision to take part in this study?
    (role of family/friends or healthcare professionals?)
- Why did you decide to take part?
  - What were your expectations of entering a research study?
  - What do you generally use a computer for?
- Where do you use a computer?
  - Have you ever used the internet for a health-related reason?
  - How have you found the information about the study?
    - **The InterSPACE self-management programme**- *have access to a computer to look at website during discussion if necessary.*
- What did you hope to get out of the programme?
  - - - Is that what happened?
  - How did you feel about the introduction you had to the online programme?
    - Was it sufficient?
- How did you get on using the online programme?
  - Did you have problems navigating the website?
  - How did you find the learning material?
  - Did you use the video conferencing option?

*If you did how useful was it?
 Is there anything you would change about it??*

- - How did you feel about the exercise programme?
  - How did you get on following it at home?
  - How did you set yourself goals, if at all?
- What did you enjoy?
- Have you had any support during the programme? (healthcare professionals/ friends/family)
  - Has anyone helped you with the programme?
  - Would you have liked any more/different support?
  - Did you contact a healthcare professional whilst on the programme? Why?
    - Has the programme influenced how you manage your condition?

Contd…..

- - - Have you had an exacerbation or felt unwell since starting the programme?
      - If **yes**, how did you manage this?
      - What did you do?
    - Will you continue what you have learnt during the programme?
    - If **yes**, how will you do this?
      What will help you do this?
      Will you continue to use the website
    - If **no**, why is this?
      - **Assessments/Outcome measures**
- You completed questionnaires at the beginning and end of the programme, how did you feel about this?
  - How suitable do you think they were?
- **Study overall**
  - How do you feel about the whole experience of taking part in a research study?
  - What could be improved? (Website/conduct of trial/timing)
  - Any advice for us in developing this online programme for other patients in the future?
  - Have you thought about dropping out from the study?
    - If **yes**, what caused this/what stopped you from withdrawing?
    - If **no**, has anything helped you stay on the study?
      - **End of Interview**
        - I have asked all the questions I would like to know.
        - Do you have anything you feel like you would like to add?
        - Thank you very much for all your time and comments.
        - I’ll turn off the Dictaphone now.
