# Supplementary material for: Web-Based Self-management Program (SPACE for COPD) for Individuals Hospitalized With an Acute Exacerbation of Chronic Obstructive Pulmonary Disease: Nonrandomized Feasibility Trial of Acceptability
Source: JMIR Mhealth Uhealth. 2021 Jun 11;9(6):e21728. doi: 10.2196/21728 (PMC8235284; doi:10.2196/21728)
Supplement: Multimedia Appendix 2 [file mhealth_v9i6e21728_app2.doc]

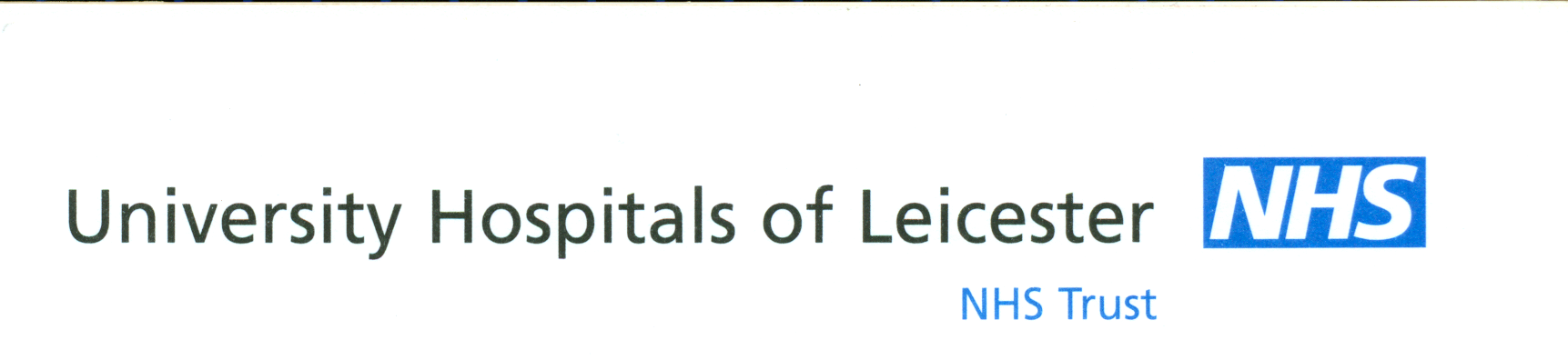


Glenfield Hospital

Groby Road

Leicester

LE3 9QP

Tel: 0300 303 1573

Fax: 0116 258 3950

Minicom: 0116 287 9852

**Follow-up Interview – Topic Guide for NON-COMPLETERS**

**InterSPACE – COPD Self-Management Programme**

InterSPACE: Feasibility of an integrated Telehealth and Self- Management programme for individuals hospitalised with an exacerbation of COPD.

- **Gain consent and introduction**
  - *Introduce you, name and role*
  - You have consented to take part in an interview which is an informal chat with me. We will be discussing your participation in the interSPACE study and your experiences of the web-based programme you have been using. It is an opportunity for you to tell me anything that you think is relevant.
  - Do you have any questions?
  - Are you happy for me turn on the Dictaphone to record the interview as it takes place?
  - For the benefit of the tape it is …day…..time and present there is me, …… (the patient)
    and …….
- **Withdrawal**
  - If anything we speak about today does make you feel uncomfortable you are free to
    not answer a particular question, request for the recorder to be switched off to
    resume the interview after a short break or you can ask to terminate the interview all
    together at any point.

Contd….

- **Participation in the InterSPACE study**
  - What influenced your decision to take part in this study?
    (role of family/friends or healthcare professionals?)
- Why did you decide to take part?
  - What were your expectations of entering a research study?
  - What do you generally use a computer for?
- Where do you use a computer?
  - Have you ever used the internet for a health-related reason?
  - How have you found the information about the study?
    - **The InterSPACE self-management programme**- *have access to a computer to look at website during discussion if necessary.*
- You completed questionnaires at the beginning of the programme, how
   did you feel about this?
- How suitable did you think the questionnaires were?
- What did you hope to get out of the web-based programme?
  - Is that what happened?
- How did you feel about the introduction you had to the web-based programme?
  - Was it sufficient?
- How did you get on using the programme?
  - Did you have problems navigating the website?
  - How did you find the reading material?
  - How did you feel about the exercise section of the programme?
  - How did you get on following the programme at home?
  - How did you set yourself goals, if at all?
  - Did you use the video conferencing option?

*If you did, how useful was it?*

- - - - - What did you enjoy?
    - Have you had an exacerbation or felt unwell since starting the programme?
      - If **yes**, how did you manage this?
      - What did you do?

Contd…..

- Have you had any support during the programme? (healthcare professionals/ friends/family)

Would you have liked any more/different support?

- - - Is there anything that you learned from the web-based programme that you would like to continue?
  - Would you like to use the programme again in the future?
    - Would you recommend the programme to other patients?
    - If **yes**, why?
    - If **no**, why?
      - **Withdrawing from Programme**
  - What influenced your decision to stop taking part in the study?
    - - - Did you talk to anyone else before making your decision?
  - What could be done differently?
  - Would could have helped you to stay on the study?
  - Would you get involved with research like this in the future?
    - - **End of Interview**
        - I have asked all the questions I would like to know.
        - Do you have anything you feel like you would like to add?
        - Thank you very much for all your time and comments.
        - I’ll turn off the Dictaphone now.
